# Can Gentisic Acid Serve as a High-Performance Antioxidant with Lower Toxicity for a Promising New Topical Application?

**DOI:** 10.3390/life14081022

**Published:** 2024-08-17

**Authors:** Luciano B. Cunha, Eloah D. Lepore, Camila Cristina B. Medeiros, Rodrigo Sorrechia, Rosemeire C. L. R. Pietro, Marcos A. Corrêa

**Affiliations:** School of Pharmaceutical Sciences, São Paulo State University (UNESP), Araraquara 14800-903, Brazil; luciano.b.cunha@unesp.br (L.B.C.); eloah.drudi@unesp.br (E.D.L.); camila.medeiros@unesp.br (C.C.B.M.);

**Keywords:** gentisic acid, 2,5-dihydroxybenzoic acid, antioxidant, antimicrobial, cytotoxic, *Staphylococcus aureus*, *Escherichia coli*, *Candida albicans*, *Cutibacterium acnes*, DPPH, NRU, topical administration

## Abstract

Gentisic acid (2,5-dihydroxybenzoic acid) is primarily found naturally in plants and has demonstrated a significant range of biological activities; however, its efficacy and safety as a topical application ingredient are not yet well established. Thus, the compound’s potential antioxidant and antimicrobial properties were evaluated for efficacy, while the cytotoxicity was evaluated for safety. The antioxidant activity, measured by the DPPH kinetic method, showed an Efficiency Concentration (EC_50_) of 0.09 with an antioxidant reducing power (ARP) of 11.1. The minimum inhibitory concentration (MIC) against *Staphylococcus aureus* was 4.15 mg/mL, *Escherichia coli* was 4.00 mg/mL, *Candida albicans* was 3.00 mg/mL, and *Cutibacterium acnes* was 3.60 mg/mL, and the MIC for *C. acnes* has remained unpublished until now. The substance showed low cytotoxicity by the neutral red uptake (NRU) methodology against HaCat, HDFa, and HepG2 cells at concentrations of up to 10.0, 7.3, and 4.0 mM, respectively, also representing unpublished data. This evidence demonstrates gentisic acid as a promising active substance for skin topical application in the cosmetic or pharmaceutical industry.

## 1. Introduction

Gentisic acid (GA) is a natural compound widely distributed in plants as a specialized or secondary metabolite, such as *Gentiana* spp., *Citrus* spp., *Helianthus tuberosus*, and *Hibiscus rosa-sinensis.* It is found as part of the plant’ defense system in a free or conjugate form. Currently, its role in the immune system is still under investigation, although some researchers have pointed out functions such as immune response modulation, where the free and associated forms of GA have its concentration increased 100-fold when a vineyard is under a Phytoplasmas attack [1,2].

The unconjugated GA concentration in some plants has been reported, for example, in *Jatropha cinerea* and *Jatropha cordata* leaves at 634.5 and 864.6 mg/kg, respectively [3], in *Vitis vinifera (Merlot)* seeds at 23.38 mg/kg [4], in *Melissa officinalis* at 21 mg/kg, and in *Salvia officinalis* at 24 mg/kg [5].

**Figure 1:**
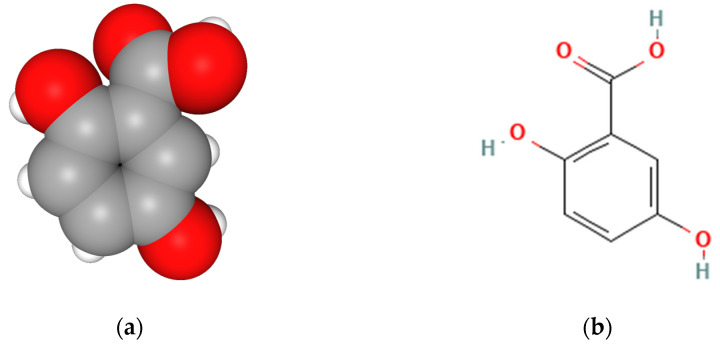
Gentisic acid (**a**) 3D space-filling molecular model and (**b**) structural formula [23].

GA is classified as having a phenolic acid in the benzene ring, and a carboxyl and two hydroxyl groups at the two and five positions. Its molecular model and structural formula are shown in Figure 1a,b. This substance has beneficial effects on human health, such as antioxidant [6,7,8], anti-inflammatory [9], anti-rheumatic [10], antiparkinsonian [11], and anti-diabetic [12,13] activities. It has shown efficacy in preventing dysfunction in the transition from hypertrophy to heart failure [14], as well as antitumor and anticancer activity in the brain [15], glial [16,17], breast [18,19,20], and skin [21]. In addition, it enhances the skin healing process, activating the pathway associated with ERK1/2 via phosphorylation in the mitogen-activated protein (MAP) kinase pathway and increasing the proliferation of keratinocytes [22].

The human skin is the largest organ in the human body; representing 5 to 16% of the weight, it envelops the entire body and performs a variety of functions, where the primary one is to serve as a protective barrier between the internal and external environments [24,25,26]. Furthermore, the skin microbiota is made of a diverse array of microorganisms that inhabit human skin. The relationship between microorganisms and humans has generated a process of mutual co-evolution, so it is expected that the relationship will be beneficial for humans and their microbiota, given that, over time, the microbiota has assumed essential functions in the physiology of the skin [27,28,29].

Researchers have dedicated themselves to understanding the skin microbiota further, given its growing importance. Evidence indicates that the type of birth, whether normal or cesarean, as well as behavioral issues, location, and other variables can impact the microbiota. It is known that the number of symbiotic microbial beings exceeds the number of human cells by at least 10 times, with an estimated 100 trillion microorganisms [30,31,32]. Consequently, the investigation of topical active ingredients should include the impact on the skin microbiota. This knowledge will support the development of products that are as minimally disruptive to the skin microbiota, while also allowing for the identification of potential antibiotic agents, since microbiota microorganisms can become pathological.

In this way, this study presents the gentisic acid antioxidant behavior via a kinetic method, the minimum inhibitory concentration (MIC) against *Staphylococcus aureus*, *Escherichia coli*, *Candida albicans*, and *Cutibacterium acnes* (formerly *Propionibacterium acnes*) [33], and the cytotoxicity against HaCat, HDFa, and HepG2 cells using neutral red uptake (NRU) methodology.

## 2. Materials and Methods

### 2.1. The Kinetic Method for Antioxidant Potential

The methodology employed was based on that of Brand-Williams, Cuvelier, and Berset [34], using the photometric reading over the time of the 1,1-diphenyl-2-picrylhydrazyl radical (DPPH) oxidation in HPLC-grade methanol, against the antioxidant samples—gentisic acid (GA) or ascorbic acid (ASC). The reagents DPPH, GA, and ASC were supplied from Sigma Aldrich, St Louis, MO, USA, and HPLC-grade methanol was from Êxodo, Sumaré, Brazil.

#### 2.1.1. DPPH Calibration Curve

The DPPH 80 µM methanolic solution was prepared through the analytical weighing of the DPPH radical and the subsequent dilution in a volumetric flask. This solution was repeatedly diluted until 60, 40, 20, 5, 2.5, and 1.5 µM concentrations. All solutions were prepared on the same day as the experiment. A standard quartz cuvette with an optical length of 10 mm was employed to measure the absorbance in a BMG Nano spectrophotometer configured in scan mode with a 1 ηm of resolution from 220 to 1000 ηm, high sensitivity, 100 shots per sample, and absorption reading mode. The reference solution was HPLC-grade methanol without additions. The readings were conducted in a dark environment, at approximately 20 °C to obtain the absorption spectrum for each solution described above. The absorbance at a wavelength of 515 ηm was correlated with the respective concentration. The least squares method was employed to find the equation and calibration curve fitting parameters.

#### 2.1.2. DPPH Radical Scavenging Kinetic Curve

The data to construct a response surface correlating the DPPH absorbance versus wavelength versus time were collected by adding 2000 µL of DPPH at 60 µM to the cuvette and performing the initial reading. This was then followed by a rapid addition of 200 µL of the antioxidant solution under study (GA or ASC). The second reading was conducted one minute after the initial reading. Subsequent readings were conducted at one-minute intervals until the end of the analysis time. All readings were performed using the same spectrophotometer parameters as outlined in Section 2.1.1. This procedure was repeated for each methanolic solution of antioxidants in different concentrations. Gentisic acid concentrations evaluated were as follows: 170, 150, 100, 50, and 30 µM, and ascorbic acid: 250,000, 180,000, 120,000, 60,000, 40,000, 4000, 800, 400, 160, 120, 100, 80, 56, 20, and 8 µM. The concentration of DPPH was calculated using the parameters of the linear adjustment of the calibration curve, as described by the following Equation (1):

C_DPPH_ = (A + b)/a (1)

where “A” is the absorbance at a wavelength of 515 ηm; and the a and b values represent the calibration curve angular and linear coefficients, respectively.

The percentage of radical inhibition or radical scavenging activity (RSA) was calculated by Equation (2):

RSA = (A_initial_ − A_current_)/A_initial_ × 100% (2)

where RSA is the percentage of free radicals inhibited at the time of testing; A_initial_ and A_current_ are the absorbances of the solution at the initial moment and the test moment, respectively.

The antioxidant molar ratio, also known as the Efficiency Concentration (EC), a dimensionless number, was calculated according to Equation (3):

EC = Q_AO_/Q_DPPH_ (3)

where Q_AO_ and Q_DPPH_ are the molar quantities of antioxidant substances and DPPH at the initial moment of the chemical reaction. EC_50_ refers to 50% of radical inhibition.

The antioxidant reducing power (ARP) is the inverse of EC, Equation (4). The antioxidant stoichiometric value is defined as twice the EC_50_.

APR = 1/EC(4)

### 2.2. In Vitro Cytotoxic Potential through Neutral Red Uptake (NRU)

The cytotoxic potential was determined in metabolically incompetent human keratinocytes (HaCat) cultured in Minimum Essential Media (MEM) for human dermal fibroblasts (HDFa) and human hepatocarcinoma (HepG2) cultured in Dulbecco’s Modified Eagle Medium (DMEM). The cell strains and mediums were provided by Life Technologies, Brazil, and the assay followed ISO 10993-5 standards [35].

In brief, the mediums were supplemented with 10% fetal bovine serum and the antibiotics penicillin (100 U/mL) and streptomycin (0.1 mg/mL). The cultures were maintained at 37 ± 2 °C in a 5% CO_2_ atmosphere. Once the cells reached 80 to 90% confluence, they were trypsinized, centrifuged at 1200 rpm for 3 min, and then seeded in 96-well plates. The trypsin was neutralized with the respective medium with fetal bovine serum, and the cell concentration for the assay was normalized at 1.0 × 10^6^ cells/mL.

The plates were incubated for 24 h to allow for cell adhesion. Soon after, 100 µL of sodium lauryl sulfate was added as a positive control. The medium, depending on the cell line, with the supplementation of 5% fetal bovine serum and antibiotics, was used as a negative control. The remaining wells were added to the samples at the concentrations to be evaluated, and the plate was incubated again for 24 h. After incubation, the plate was gently inverted to remove the culture medium and washed with 150 µL of PBS.

Following this step, 100 µL of the medium with neutral red at 40 µg/mL was added to each well and incubated again for 3 h. After the short incubation, the suspension from each well was removed by inverting the plate again and washing it with 150 µL PBS. The desorption solution, 150 µL of acetic acid, was added and subjected to orbital shaking for 10 min. The absorbance at 540 ηm was measured using a spectrophotometer equipped with a microplate reader.

### 2.3. Determination of Antimicrobial Activity

The Minimum Inhibitory Concentration (MIC) was determined using a method based on Kalinowska’s research group at Bialystok University of Technology in Poland [36] with some key modifications. In summary, the method involved the dilution of GA solutions with a warm Mueller–Hinton agar (MHA) heated up until approximately 48 °C, where the GA solutions and MHA were mixed directly in the test plate for the subsequent microorganism inoculation.

A sterile flat-bottom 24-well plate with lid (Costar™) was used, with twelve wells designated for MHA with increasing concentrations of GA, another eight wells for increasing concentrations of the antimicrobial control, and finally the remaining four wells for test plate controls—the sample, the MHA, the microorganism, and the sample diluent.

The well with the lowest concentration that inhibited the growth of the microorganism represents the MIC for both the sample and the antimicrobial control. The arithmetic mean of the independent triplicates of each tested substance, controls, and microorganisms was considered the MIC.

Three solutions of gentisic acid at concentrations of 60, 45, and 30 mg/mL were prepared using demineralized water with the addition of 5% dimethyl sulfoxide (DMSO) in a Falcon tube. The solutions were submitted to vigorous agitation for 40 min in a vortex shaker and were designated as SP1, SP2, and SP3, respectively.

The antimicrobial control was prepared in an aqueous solution using sterile demineralized water as a diluent, with the following concentrations: Ampicillin 0.2 and 0.05 mg/mL for *S. aureus* and *E. coli*; Ampicillin 0.005 and 0.0014 mg/mL for *C. acnes*, and Fluconazole 0.08 and 0.0016 mg/mL for *C. albicans*. The highly concentrated solution of each antibiotic control was named AP1, while the solution with the lower concentration was named AP2.

The 24-well plate was prepared by adding between 1.80 and 1.92 mL of MHA to each well, along with between 0.08 and 0.20 mL of the samples, ensuring that the final volume of each well was 2.00 mL. The procedure was conducted by applying heated agar at approximately 48 °C, using a variable volume pipette with a sterile tip. The GA or the antimicrobial solutions were then added to the agar and homogenized by repeated suction and dispensing of the mixture (four times).

The microbial solutions of *Staphylococcus aureus* ATCC 6538, *Escherichia coli* ATCC 10536, and *Cutibacterium acnes* ATCC 6919 were standardized at a 0.5 McFarland equivalent to 1.5 × 10^8^ CFU/mL and subsequently diluted to 1.5 × 10^6^ CFU/mL. *Candida albicans* ATCC 10231 were counted in a Neubauer chamber and standardized at 2.0 × 10^4^ CFU/mL. These microbial solutions were then inoculated onto the solidified agar using a sterile swab in each well that should receive the microorganisms. The plate was incubated for 24 ± 0.5 h at 35.5 ± 2 °C for *Candida albicans*, at 37 ± 2 °C in the case of *Staphylococcus aureus* and *Escherichia coli,* and 48 ± 1 h at 37 ± 2 °C in an anaerobic chamber for *Cutibacterium acnes*.

**Figure 2:**
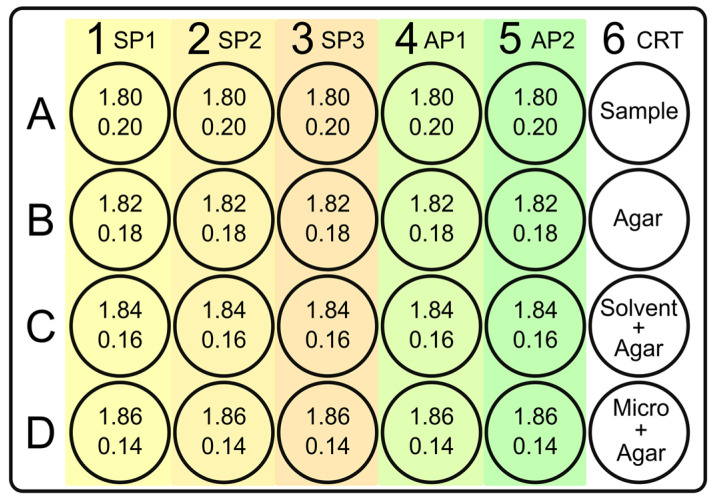
Map of the 24-well plate as an example. The first numerical line in each well represents the volume of agar to be added in mL. The second numerical line represents the volume of active solution to be applied. The substance to be assessed is applied in columns 1 to 3, which are shaded in yellow to orange. The control antibiotic is applied in columns 4 and 5, green shaded. Column 6 is reserved for the experiment quality controls. SP1, SP2, and SP3 denote the specific solutions of the tested substance assigned to each column. Meanwhile, AP1 and AP2 correspond to the antimicrobial solutions designated for the same purpose.

To ensure the efficacy of the experiment, a plate well map was created for each condition (microorganism, analyte, and antimicrobial). This is exemplified in Figure 2.

### 2.4. Data Analysis

The statistical significance adopted for the entire study was 95%. The calculation was supported by JASP 0.17.1 [37], and the data were plotted using either Microsoft Excel™ version 365 or MARS™ Data Analysis 3.01 R2.

## 3. Results

### 3.1. Antioxidant Potential

The chemical reaction medium in a DPPH assay can be represented in terms of photometric absorption between the radical and the antioxidant, in this case, gentisic acid or ascorbic acid, by a response surface involving the absorption level versus the wavelength versus time. Once the surface elevation demonstrates the amount of radicals in their characteristic wavelength interval, consequently, a decrease in the surface level will show the amount of radicals being sequestered by the other reactant.

**Figure 3:**
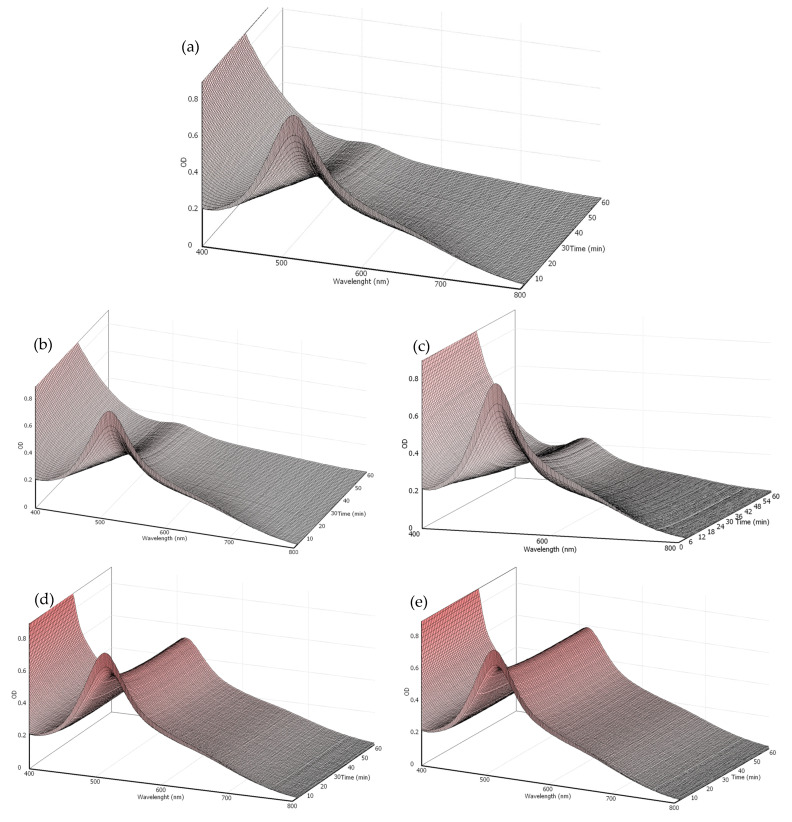
The surface response showing the wavelength vs. time vs. absorption behavior for each initial concentration of gentisic acid, (**a**) 170 µM, (**b**) 150 µM, (**c**) 100 µM, (**d**) 50 µM, and (**e**) 30 µM.

The initial set of measurements, taken at a GA concentration of 170 µM, is represented by the response surface in Figure 3a. The foreground region of the figure shows the initial DPPH concentration, with the depth of the image indicating a decrease in radical concentration over time. However, the lack of a stable surface at the end of the reaction period suggests that the process has not reached chemical equilibrium. Indeed, even after one hour of reaction, the steady state has not been attained. This observation will be further addressed in the text.

The analysis of lower GA concentrations, Figure 3b–e, demonstrates the concentration effect on the amount of radical scavenging. The 150 µM GA concentration, Figure 3b, exhibited a similar pattern, although with a lower degree of radical neutralization. It is noteworthy that a plateau in absorbance is created in the final minutes of the 100 µM reaction, Figure 3c. This behavior is more evident in Figure 3d, 50 µM, where a stable level of absorbance is observed, indicating that the reaction has reached its steady state. Similarly, in Figure 3e, 30 µM, the same behavior can be seen with the surface reaching a stability level at a higher absorbance, indicating that the reaction reached its equilibrium state with a smaller amount of radical sequestered, given the smaller amount of gentisic acid added.

**Figure 4:**
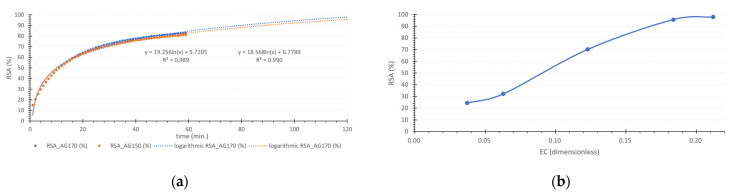
(**a**) logarithmic prediction model to estimate the RSA parameter at 120 min of reaction is related to 150 and 170 µM concentrations of gentisic acid. (**b**) The RSA is represented at a steady state as a function of Efficiency Concentration (EC).

The calibration curve, which correlates absorbance with gentisic acid concentration, yielded a slope of 0.0127 and an r^2^ value of 0.9995. This slope was applied in Equations (1) and (2) to determine the Radical Scavenging Activity (RSA) for each reaction time. While certain researchers recommend allowing reactions to reach a steady state before conducting antioxidant calculations [38,39,40,41], others accept methods that do not require this condition [42,43,44,45,46,47]. In adherence to the methodology described in Section 2.1, which recommends a steady state, a logarithmic prediction model was employed (as shown in Figure 4a) to estimate the equilibrium RSA for GA concentrations of 150 and 170 µM, which had not reached stability at the time of the assay. Those estimations were applied into Equation (3), enabling the generation of a graph that illustrates RSA at steady state versus the Efficiency Concentration (EC), as depicted in Figure 4b. The graphical analysis revealed an EC_50_ of 0.09, and the subsequent use of Equation (4) resulted in an Antioxidant Reducing Power (ARP) of 11.1.

**Figure 5:**
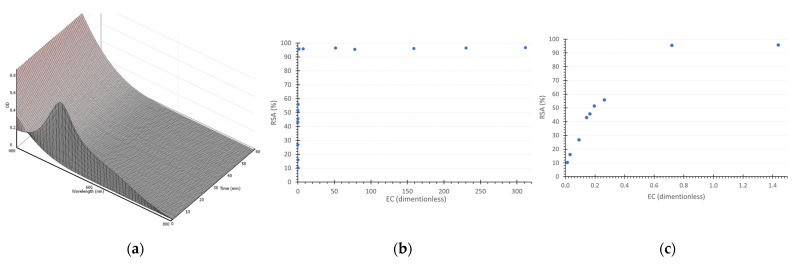
(**a**) The surface response showing the wavelength vs. time vs. absorption behavior for 180 mM of ASC, (**b**) The radical inhibition percentage for the 15 ASC concentrations assessed, (**c**) the same as (**b**), but focusing on the range of EC until 1.5, which allows the EC_50_ determination.

The same process was applied to 15 concentrations of ascorbic acid, as in Figure 5b, presenting a much faster kinetic behavior, with 180 mM as an example in Figure 5a. The ASC presented a DPPH radical inhibition limit of around 96%, focusing on the EC range until 1.5 (Figure 5c), and it is possible to notice a nonlinear relation between the RSA and EC. The same figure allows for the EC_50_ determination of 0.185 and, applying Equation (4), results in an APR of 5.4. The EC_50_ comparison between the gentisic and ascorbic acid was 0.09 and 0.185, respectively, showing gentisic acid to be around two times more antioxidant than the ascorbic acid.

### 3.2. Evaluation of Cytotoxic Potential

**Figure 6:**
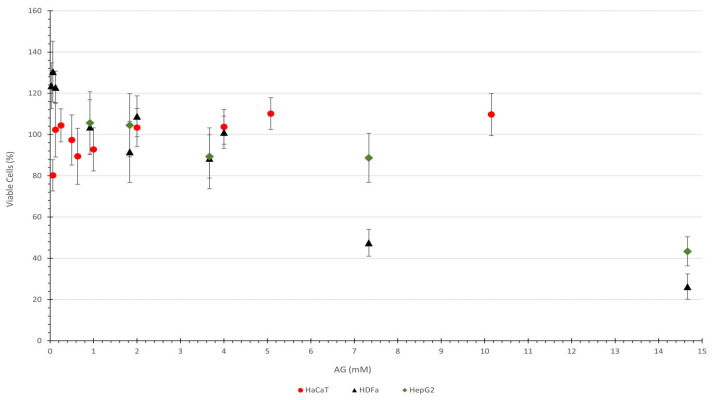
Cell viability percentage as a function of AG concentration for each cell line. Circle marks are related to HaCat, triangle to HDfa, and diamond to HEPG2. The error bar is related to the confidence interval at the 95% level.

Conducting the experiment was challenging by itself, since the GA is a polyprotic acid with a significant dissociation constant, pKa_1_ of 2.87 [48]. Achieving stability required several retests due to pH issues with the buffer solution, making these results crucial for future work. The cytotoxic potential evaluation using NRU methodology and GA was unpublished at the time of drafting the manuscript for this article. The cell viability percentages and confidence intervals of the collected data are available in Figure 6.

The HaCat cells strain presented a high viability level, around 100%, up to the maximum GA concentration of 10.15 mM. Some available data are related to the MTT methodology, which does not allow a fair comparison, but they have also found a high viability level [22].

The HDfa cell strain presented a viability level of around 125% at a low concentration of GA until 0.13 mM. The GA concentration of 7.33 mM showed a cell viability of 47.5% with a confidence interval of between 41 and 54%, including 50%. This means that the IC_50_ has a value of around 7 mM of GA. No published data were found for HDFa against GA with the NRU methodology to provide a comparison.

The final cell strain assessed was HepG2, which demonstrated high viability at concentrations up to 7.3 mM. The IC_50_ should remain at approximately 14 mM, as the next higher concentration (14.67 mM) demonstrated a viability interval of 36.3 and 50.4%. No published data were found for this specific assay. However, researchers have identified a hepatoprotective effect in the *Myrciaria dubia* extract when evaluated against HCT8 cells, which are not a human strain but derived from rats. This result was attributed to the presence of gentisic acid in this extract [49]. While not an entirely accurate comparison, this association provides a degree of rationale for the results observed here.

GA concentrations higher than 14 mM proved to be unfeasible to be assessed since there was an appearance change in the medium.

### 3.3. Assessment of Antimicrobial Potential

For all tested microorganisms, *Staphylococcus aureus*, *Escherichia coli*, *Candida albicans* and *Cutibacterium acnes*, GA presented MIC values ranging from 3.00 mg/mL to 4.15 mg/mL. Detailed data are available in Table 1.

## 4. Discussion

In the context of topical applications, GA demonstrated a robust antioxidant capacity surpassing that of ASC, a reference substance in the industry. The results presented in the antioxidant section (an EC_50_ of 0.09 and an ARP of 11.1) confirm data previously shown by Brand-Williams et al. [34]. However, a clear distinction has emerged in terms of reaction rate. It can be explained based on the differences in reduction mechanism once the ASC reaction uses electron transferring, which is faster than the transferring of a hydrogen atom used by AG [40,41]. This understanding may motivate further investigation into optimal ways to employ GA’s antioxidant potential, including potential blends with ASC. Such deeper knowledge would support the use of this compound as an antioxidant in formulations or as an active ingredient for products targeting the oxidative cascade.

Oxidative stress has been identified as a primary factor triggering skin aging [50,51]. Once the collagen degradation caused by changes in the extracellular matrix has increased, the oxidative stress would also increase, creating a feedback system [52]. The growing oxidative cascade contributes to cutaneous cancer [53] and a microbiota disorder [54], making a substance with a high antioxidant capacity an important ingredient in cosmetic or pharmaceutical formulations targeting skin protection.

This oxidative scenario above requires the assessing of whether an antioxidant agent is cytotoxic to skin cells. The current evaluation did not include all strains of cells in human skin; however, since keratinocytes constitute 90% of the epidermis and fibroblasts across most of the dermis [55], both were evaluated, suggesting that the substance is likely safe. Gentisic acid demonstrated favorable cytotoxicity results up to concentrations of 4 mM, showing no toxicity to the keratinocytes and fibroblasts. The liver cell strain evaluated here aids in predicting the potential effects if the substance reaches the circulatory system, where the liver would metabolize it. The results indicate that it should be safe for the liver at this concentration level. The NRU assay with gentisic acid faced some challenges, particularly with the GA dissociation and the buffer solution. Despite the limited data for direct comparison, these results are consistent with those from the MTT method, as shown at Section 3.2 above. Nonetheless, the variability seen with NRU and GA suggests that further improvement is necessary.

The MIC of GA against *S. aureus* (4.15 mg/mL) falls within the range of previously reported values (5 mg/mL by Kalinowska et al. [36], no activity at 1 mg/mL by Vandal et al. [56]). Similarly, the MIC against *E. coli* (4.00 mg/mL) is consistent with the published data (3 mg/mL by Kalinowska et al. [36], 5 mg/mL by Feldeková et al. [57]), while the MIC against *C. albicans* (3.00 mg/mL) is intermediate to previously reported values (5 mg/mL by Kalinowska et al. [36], 1 mg/mL by Vandal et al. [56]). However, no comparable data were found for *C. acnes* (3.60 mg/mL) in the literature.

GA, at a concentration of 4 mM (approximately 0.6 mg/mL), did not exhibit an antimicrobial effect against the microbiota representatives assessed in this study. The microbiota comprises a vast number of species, yet only four were tested here. Nevertheless, given the substantial difference between this dose and the antimicrobial range of GA (3.0 to 4.15 mg/mL), these findings suggest that GA has the potential to be considered microbiota-friendly up to a concentration of 4 mM.

At the same dose level, GA also exhibited a lack of cytotoxicity, as previously discussed. These findings support the potential of GA as a promising ingredient for topical cosmetic applications as a microbiota-friendly and antioxidant ingredient.

The study also aimed to determine if gentisic acid could be used against *Acne vulgaris* and its main associated microorganism, *C. acnes*, as this skin disease affects between 60% and 95% of the adolescent population [58]. Researchers have reported a meaningful change in the disease mechanism, suggesting that the diversity of *Cutibacterium acnes* subspecies, such as *C. acnes* ssp. *elongatum*, *C. acnes* ssp. *acnes*, *C. acnes* ssp. *defendens*, and others, secrete a larger amount of reactive and oxidative substances into the skin’s pores. This oxidative load triggers the inflammatory cascade. In healthy skin, the local microbiota does not produce oxidizing agents in such substantial amounts thus not triggering the inflammatory cascade, [59,60,61,62,63,64]. This new understanding of *Acne vulgaris* represents a promising opportunity for future research into the interaction between gentisic acid, obtained from natural sources or from synthesis, and *C. acnes* and its subspecies, given the substance’s strong antioxidant potential.

## Figures and Tables

**Table 1:** Gentisic acid minimum inhibitory concentration (MIC) ± standard deviation for each accessed microorganism, control antibiotic, and the experiment control results.

Samples/ Microorganisms	MIC			
	*Staphylococcus aureus*	*Escherichia coli*	*Candida albicans*	*Cutibacterium acnes*
GA *	4.15 ± 0.09	4.00 ± 0.69	3.00 ± 0.26	3.60 ± 0.00
Ampicillin **	<3.5 ± 0.00	14.00 ± 0.00	-	>0.5 ± 0.00
Fluconazole **	-	-	8.00 ± 0.00	-
Experiment control	Passed	Passed	Passed	Passed

* results are expressed as mg/mL; ** results are expressed as µg/mL, - not applicable.

## Data Availability

The original contributions presented in the study are included in the article, further inquiries can be directed to the corresponding author.
